# Supplementary material for: Revealing the complexity of ionic liquid–protein interactions through a multi-technique investigation
Source: Commun Chem. 2020 May 6;3:55. doi: 10.1038/s42004-020-0302-5 (PMC9814843; doi:10.1038/s42004-020-0302-5)
Supplement: Supplementary file 2 — Supplementary Information [file 42004_2020_302_MOESM2_ESM.pdf]

Supplementary Information

Revealing the Complexity of Ionic Liquid-Protein Interactions through a Multi-Technique Investigation

Liem Bui-Le[1], Coby J. Clarke[1], Andreas Bröhl[1], Alex P. S. Brogan[2], James A. J. Arpino[1], Karen M. Polizzi[1], Jason P. Hallett[1*]

[1]Department of Chemical Engineering, Imperial College London, London, SW7 2AZ, UK.

[2]Department of Chemistry, King's College London, Britannia House, London, SE1 1DB

*Correspondence to Prof. Jason P. Hallett (j.hallet@imperial.ac.uk)

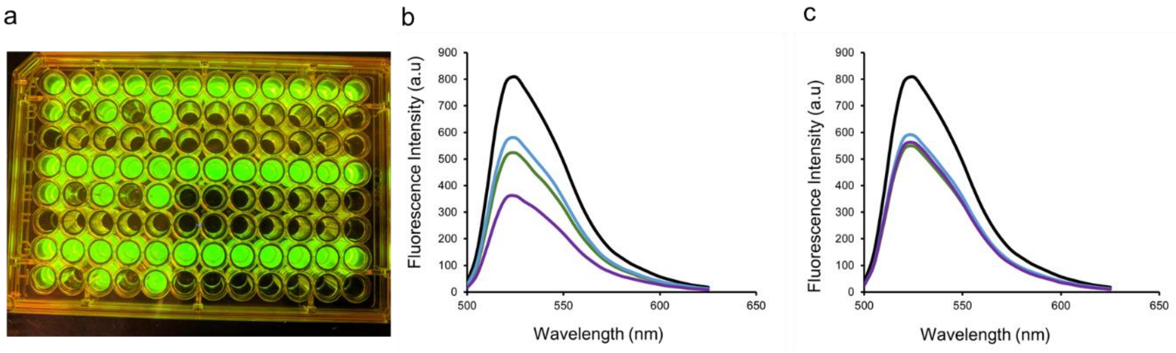

Supplementary Fig. 1: (**a**) Photo of GFP in water, and aqueous IL salt solutions showing retention of fluorescence in IL solutions (**b-c**) Fluorescence spectroscopy data for GFP dissolved in (**b**) water (black), 1 M [bmpyrr][OAc] (green), 1 M [bmpyrr][Cl] (blue), and 1 M [bmpyrr][OTf] (purple), and (**c**) water (black), 1 M [bmim][OAc] (green), 1 M [bmim][Cl] (blue), and 1 M [bmim][OTf] (purple).

Supplementary Table 1: Well plate assignments for Supplementary Fig. 1a.

	A	B	D	E	G	H
**1**	Water	1 M [emim][OAc]	Water	1 M [emim][OAc]	Water	1 M [emim][OAc]
**2**	0.1 M Tris pH 8	1 M [emim]Cl	0.1 M Tris pH 8	1 M [emim]Cl	0.1 M Tris pH 8	1 M [emim]Cl
**3**	1 M [bmpyrr][OAc]	30 wt% [emim][OAc]	1 M [bmpyrr][OAc]	30 wt% [emim][OAc]	1 M [bmpyrr][OAc]	30 wt% [emim][OAc]
**4**	1 M [bmpyrr]Cl	30 wt% [emim]Cl	1 M [bmpyrr]Cl	30 wt% [emim]Cl	1 M [bmpyrr]Cl	30 wt% [emim]Cl
**5**	1 M [bmpyrr][OTf]	30 wt% [emim][OTf]	1 M [bmpyrr][OTf]	30 wt% [emim][OTf]	1 M [bmpyrr][OTf]	30 wt% [emim][OTf]
**6**	30 wt% [bmpyrr][OAc]	-	30 wt% [bmpyrr][OAc]	-	30 wt% [bmpyrr][OAc]	-
**7**	30 wt% [bmpyrr]Cl	-	30 wt% [bmpyrr]Cl	-	30 wt% [bmpyrr]Cl	-
**8**	1 M [bmim][OAc]	-	1 M [bmim][OAc]	-	1 M [bmim][OAc]	-
**9**	1 M [bmim]Cl	-	1 M [bmim]Cl	-	1 M [bmim]Cl	-
**10**	30 wt% [bmim][OAc]	-	30 wt% [bmim][OAc]	-	30 wt% [bmim][OAc]	-
**11**	30 wt% [bmim]Cl	-	30 wt% [bmim]Cl	-	30 wt% [bmim]Cl	-
**12**	30 wt% [bmim][OTf	-	30 wt% [bmim][OTf]	-	30 wt% [bmim][OTf]	-

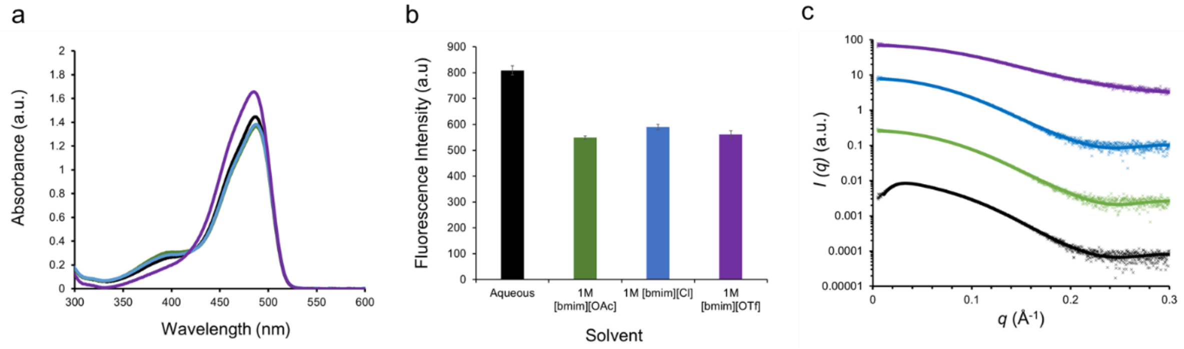

Supplementary Fig. 2: (a) UV/Vis absorbance spectroscopy data (absorbance against wavelength) showing the GFP chromophore is in its native state (**b**) Fluorescence intensity (a.u.) at 525 nm (error bars correspond to standard deviations, n = 3), and (**c**) SAXS profiles (separated for clarity) fitted with a cylinder model for GFP dissolved in water (black), 1 M [bmim][OAc] (green), 1 M [bmim][Cl] (blue), and 1 M [bmim][OTf] (purple) at 25 °C.

Supplementary Table 2: Structure dimensions determined from SAXS profiles of GFP dissolved in water, buffer, aqueous ionic liquid solutions.

Solution	Guinier Fit Diameter (Å)	Length (Å)	Diameter (Å)	Aspect Ratio (L/D)	χ2 per data points
Aqueous	39.0 ± 0.2	49.9 ± 0.19	34.57 ± 0.0	1.44	0.9
1 M [bmpyrr][OAc]	40.4 ± 0.2	52.6 ± 0.1	33.8 ± 0.0	1.56	1.3
1 M [bmpyrr][Cl]	40.0 ± 0.2	52.6 ± 0.1	34.0 ± 0.0	1.55	1.1
1 M [bmpyrr][OTf]	30.2 ± 0.2	44.3 ± 0.2	26.3 ± 0.1	1.69	1.1
1M [bmim][OAc]	39.4 ± 0.2	51.4 ± 0.1	33.9 ± 0.0	1.52	1.2
1M [bmim][Cl]	39.9 ± 0.2	52.3 ± 0.1	34.05 ± 0.0	1.54	1.0
1M [bmim][OTf]	29.2 ± 0.2	41.8 ± 0.1	24.5 ± 0.1	1.7	1.1

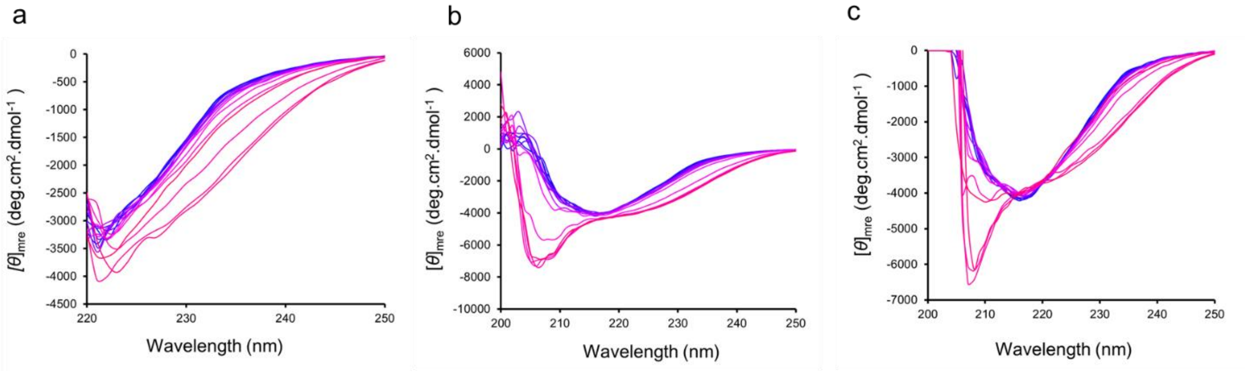

Supplementary Fig. 3: (**a-c**) Temperature dependent CD showing thermal denaturation of GFP dissolved in (**a**) 1 M [bmpyrr][OAc], (**b**) 1 M [bmpyrr][Cl], and (**c**) 1 M [bmpyrr][OTf].

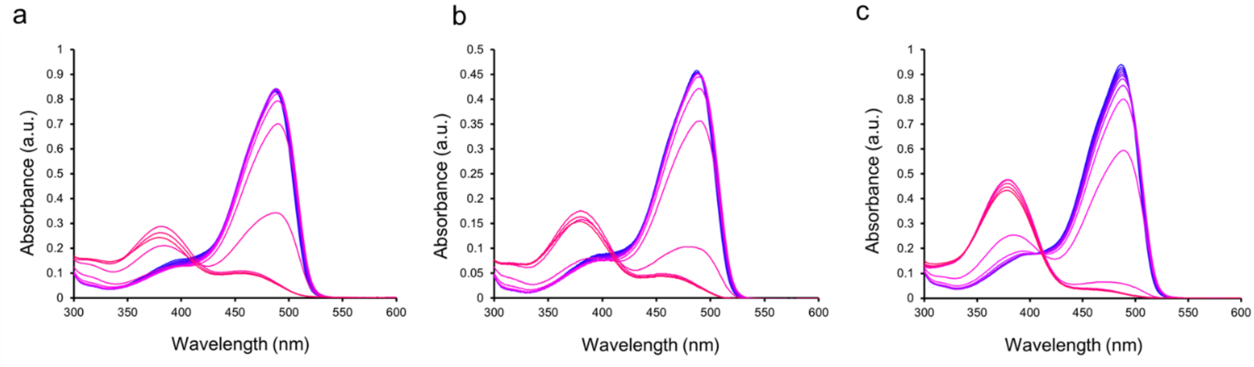

Supplementary Fig. 4: (**a-c**) Temperature dependent UV-vis spectroscopy showing thermal denaturation of GFP dissolved in (**a**) 1 M [bmpyrr][OAc], (**b**) 1 M [bmpyrr][Cl], and (**c**) 1 M [bmpyrr][OTf].

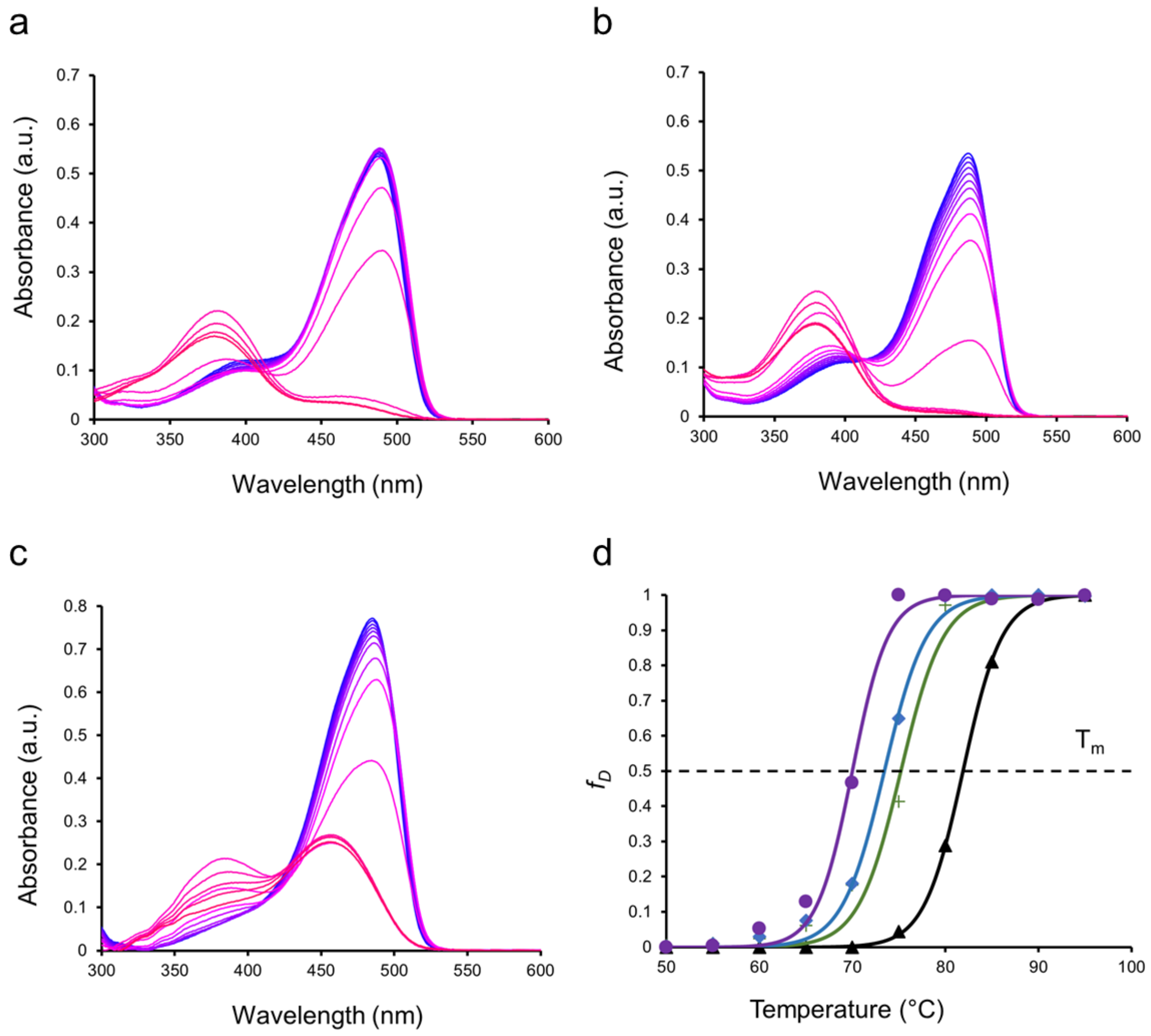

Supplementary Fig. 5: (**a-c**) Temperature dependent UV/Vis showing thermal denaturation of aqueous GFP dissolved in (**a**) 1 M [bmim][OAc] (**b**) 1 M [bmim][Cl], and (**c**) 1 M [bmim][OTf]. (**d**) Plots of fraction denatured as calculated using a two-state model of denaturation from UV/Vis spectroscopy, determined from (**a-c**), for GFP in pure water (black triangles), 1 M [bmim][OAc] (green pluses), 1 M [bmim][Cl] (blue diamonds), 1 M [bmim][OTf] (purple circles). Solid lines represent data is fitted with sigmoid.

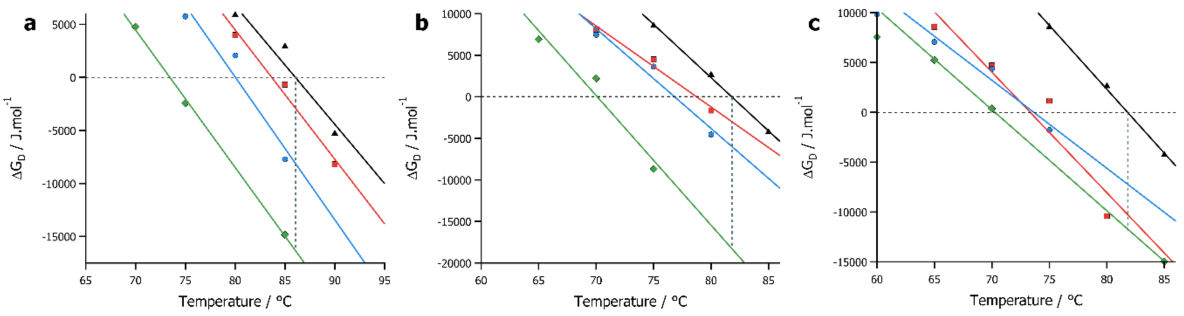

Supplementary Fig 6: Plots of free energy of denaturation calculated from temperature dependent CD measurements (**a**) and UV/vis measurements (**b-c**) for GFP in aqueous solution (black triangles) and 1 M ionic liquid solutions with the anions; [OAc$^-$] (red squares), Cl$^-$ (blue circles), and OTf$^-$ (green diamonds) and the cations; bmpyrr (**a-b**) and bmim (**c**).

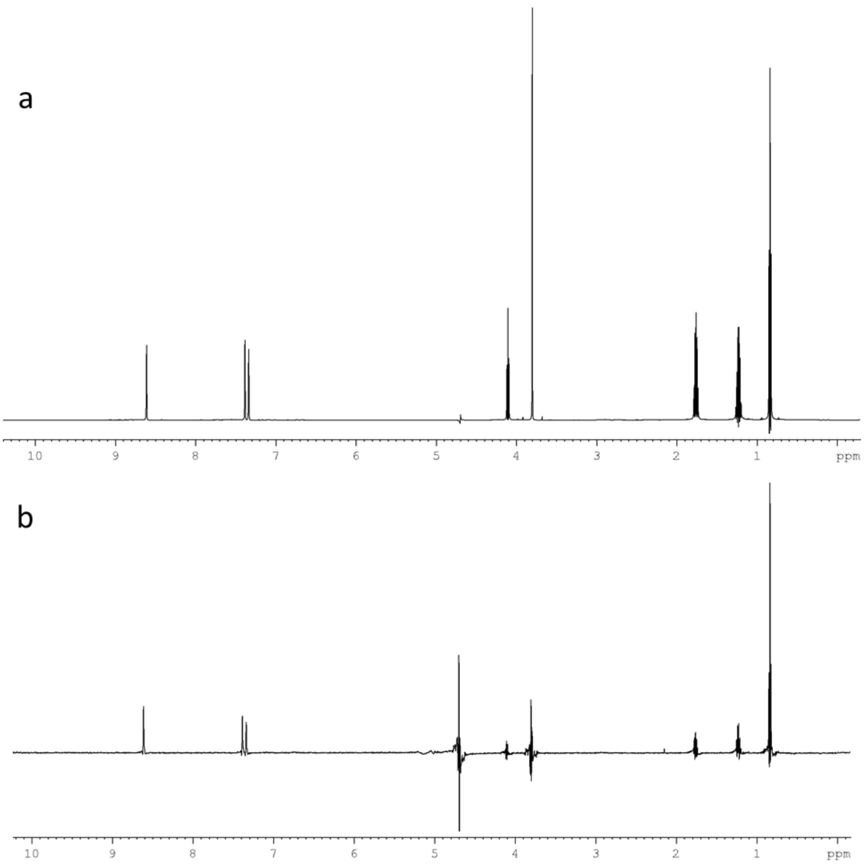

Supplementary Fig. 7: ^{1}H NMR (**a**) and STD-NMR (**b**) of 1 M [bmim][Cl] and GFP with IL:protein ratio of 1000:1 (IL concentration = 50 mM, GFP concentration = 50 μM) in D_2O.

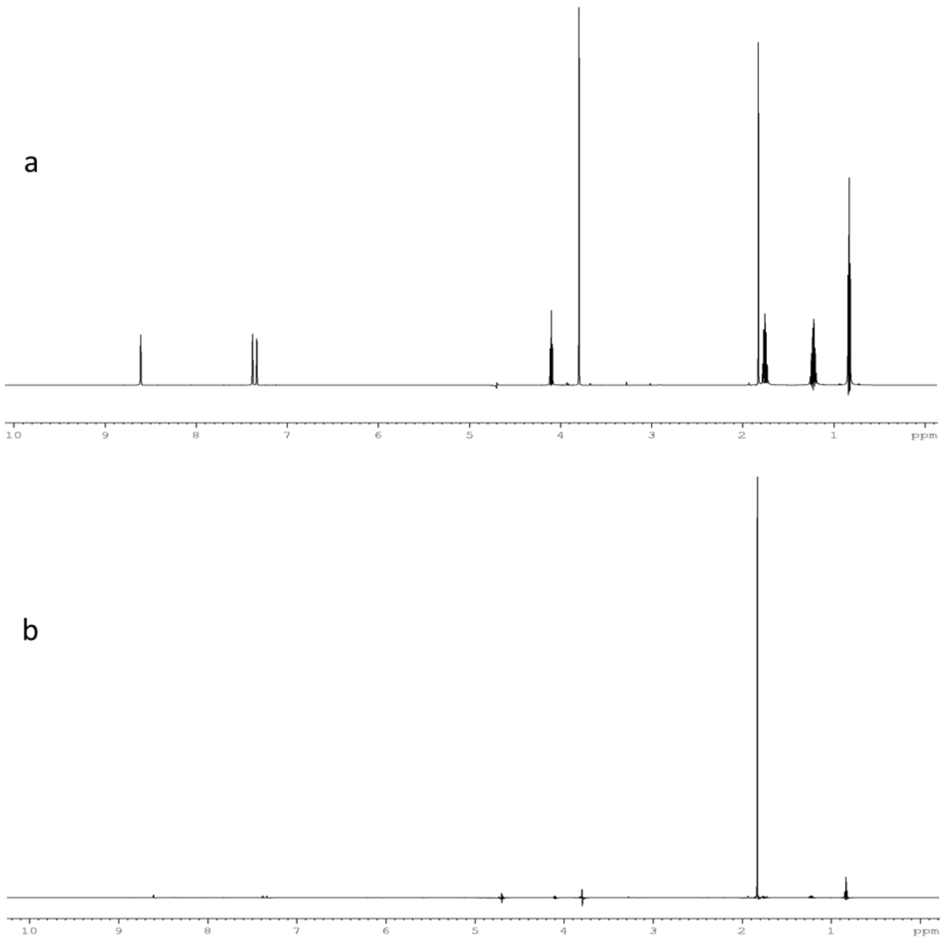

Supplementary Fig. 8: ^{1}H NMR (**a**) and STD-NMR (**b**) of 1 M [bmim][OAc] and GFP with IL:protein ratio of 1000:1 (IL concentration = 50 mM, GFP concentration = 50 µM) in D_2O.

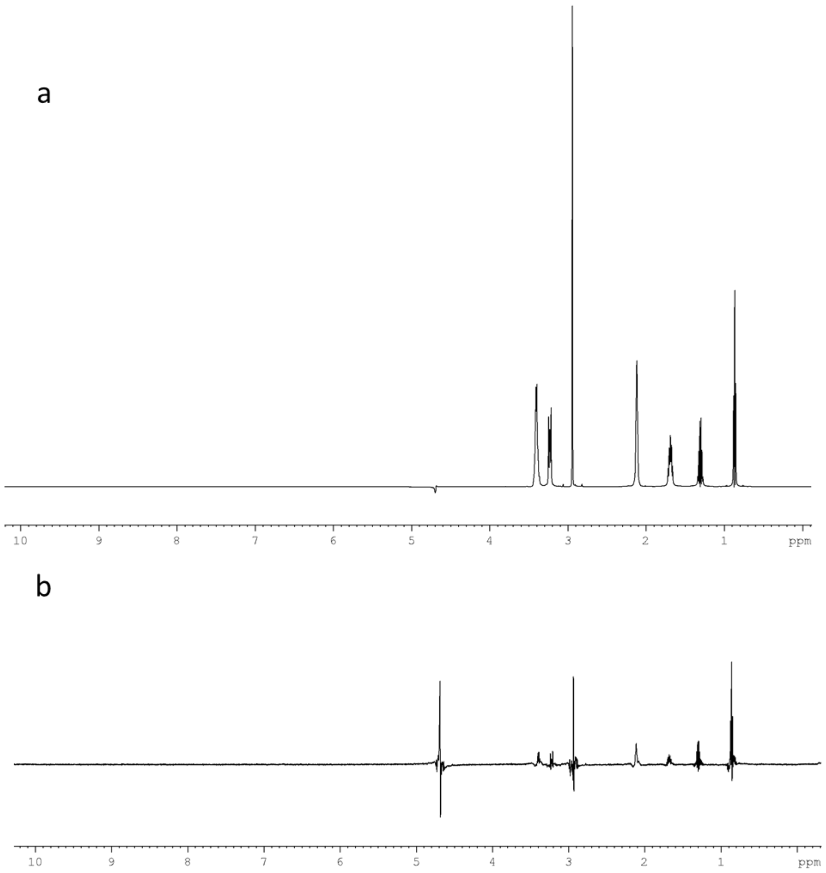

Supplementary Fig. 9: ^{1}H NMR (**a**) and STD-NMR (**b**) of 1 M [bmpyrr][OTf] and GFP with IL:protein ratio of 1000:1 (IL concentration = 50 mM, GFP concentration = 50 µM) in D_2O.

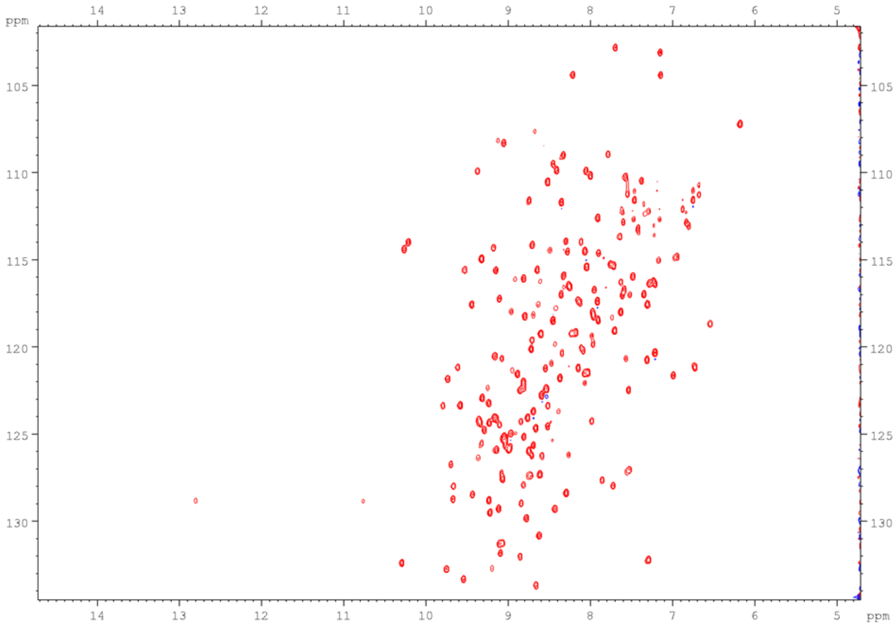

Supplementary Fig. 10: ^{1}H-^{15}N HSQC of GFP at pH 7.2 (GFP concentration = 50 µM) in D_2O.

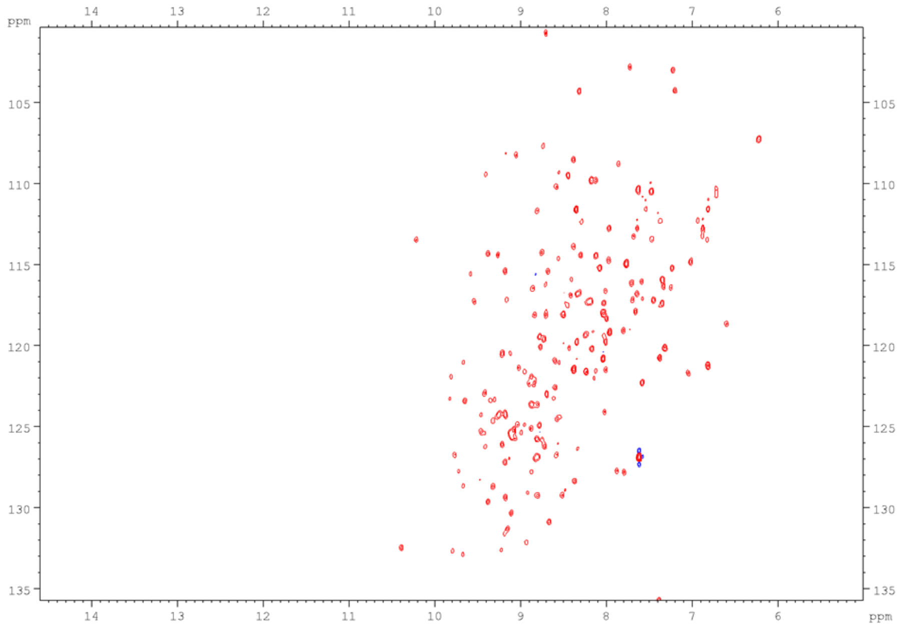

Supplementary Fig 11: ^{1}H-^{15}N HSQC of GFP in 1 M [bmim][Cl] (GFP concentration = 50 µM) in D_2O.

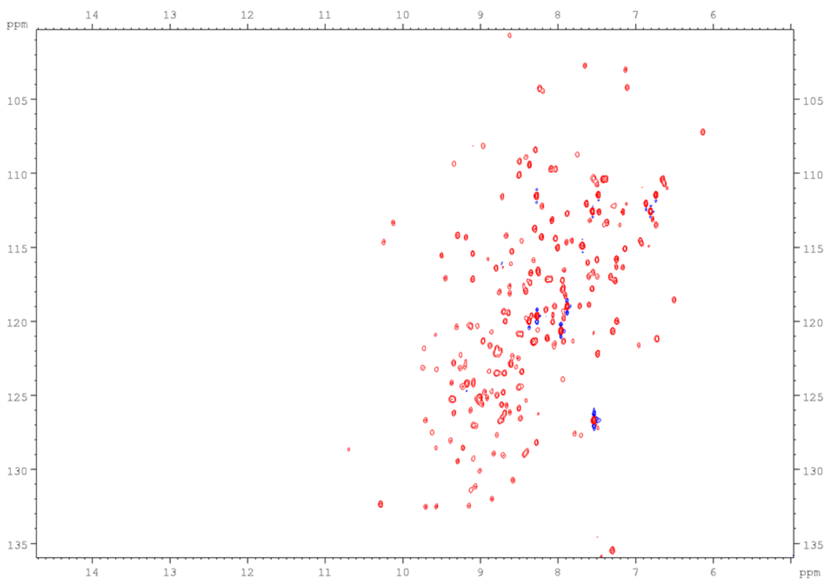

Supplementary Fig 12: ^{1}H-^{15}N HSQC of GFP in 1 M [bmim][OAc] (GFP concentration = 50 µM) in D_2O.

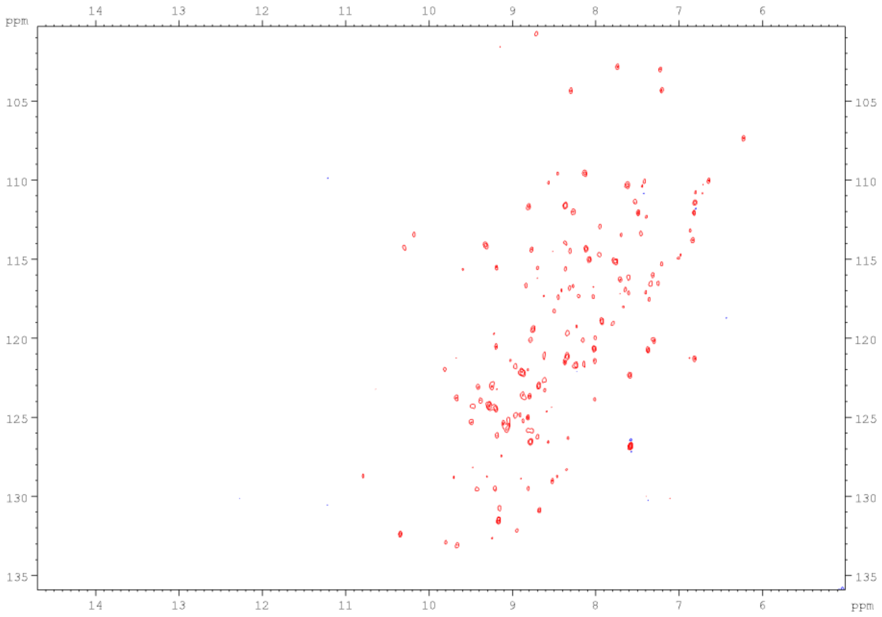

Supplementary Fig. 13: ^{1}H-^{15}N HSQC of GFP in 1 M [bmpyrr][OTf] (GFP concentration = 50 µM) in D_2O.
